# “Anything can happen”: experiences of people using opioids in a xylazine market

**DOI:** 10.1186/s12954-025-01275-z

**Published:** 2025-07-31

**Authors:** Megan K. Reed, Karla Martin González, Traci C. Green, Tracy Esteves Camacho, Rose Laurano, Frida Clark-García, Catalyst Geraci, Ava Kane, Kristin L. Rising

**Affiliations:** 1 Center for Connected Care, Thomas Jefferson University, 1015 Walnut Street, Curtis Building, Suite 704, Philadelphia, PA 19107 USA; 2 Department of Emergency Medicine, Sidney Kimmel Medical College, Thomas Jefferson University, Philadelphia, PA USA; 3 College of Population Health, Thomas Jefferson University, Philadelphia, PA USA; 4 Opioid Policy Research Collaborative, Brandeis University, Waltham, MA USA; 5 COBRE on Opioids and Overdose at Rhode Island Hospital, Providence, RI USA; 6 Department of Emergency Medicine, The Warren Alpert Medical School of Brown University, Providence, USA; 7 Department of Epidemiology, Brown School of Public Health, Providence, RI USA; 8 Project SAFE, Philadelphia, PA USA

**Keywords:** Community-based participatory research, People who use opioids, Xylazine, Harm reduction

## Abstract

**Background:**

Xylazine is associated with a number of negative health outcomes, such as severe wounds and withdrawal. As xylazine increasingly emerges in drug markets across the United States, there is an urgent need for a national response to reduce related harm. As such, healthcare systems, harm reduction organizations, and community members continue to work to identify and develop the best approaches for harm reduction interventions to address xylazine challenges. This study engaged people who use opioids (PWUO) in Philadelphia to explore current experiences with and responses to xylazine adulteration.

**Methods:**

From January to February 2024, we conducted semi-structured interviews with PWUO in the Kensington neighborhood of Philadelphia to explore their perceptions of and experiences with xylazine. We used a Community-Based Participatory Research (CBPR) framework to include people with living experiences of xylazine use in the research process. The interviews were audio-recorded, transcribed, and coded using content analysis to identify themes.

**Results:**

Among 40 participants, 50% were male, 55% White, 32% Latino, half completed high school and 40% were unhoused. Ages ranged from 25 to 57 years old. All reported using xylazine and fentanyl, and a majority (75%) had experienced an overdose in their lifetime. More than half (57.5%) reported injecting as a preferred method for mode of administration. Participants reported profound impacts from xylazine on themselves and the broader community of PWUO in Philadelphia. Conversations primarily centered on four domains of concern: wounds, withdrawal, safety concerns from unconscious sedation, and safety concerns from mobile sedation.

**Conclusions:**

Xylazine had many negative impacts on PWUO, including severe wounds, withdrawal, and sedation, with sedation resulting in increased risk of robbery, physical or sexual assault, or accidents while sedated but mobile. Individual attempts to reduce these xylazine-related harms are insubstantial when placed against structural factors driving these risks. Increased investment in wound care supplies and streetside wound care nursing by trauma-informed clinicians who will not further stigmatize people in need of care may be beneficial. In addition, higher-level policy and programmatic changes, i.e., updating clinical procedures to treat withdrawal, access to housing, and lower barrier treatment facilities, are needed to reduce the harms associated with xylazine use most effectively.

## Introduction

The United States is currently in the fourth “wave” of the overdose crisis. The first wave was marked by overdoses primarily attributable to prescription opioids. The second wave saw a transition in overdose deaths due to heroin. In 2013, fentanyl began to replace heroin and around 2015, deaths from opioids with stimulants present began to increase significantly [1]. During these transitions, the addition of adulterants to the opioid drug supply accelerated [2]. Recently, the prevalence of xylazine, or “tranq”, in the fentanyl supply has significantly increased in the mainland United States [3]. Xylazine is commonly used in veterinary medicine for sedating large animals and is not approved for human use [4]. It has life-threatening effects in humans, including central nervous system and respiratory depression, significant slowing of the heart rate, and low blood pressure [5]. Further, xylazine is associated with an increased risk for skin ulcerations, exacerbates opioid withdrawal with severe agitation, hypertension, and insomnia [6, 7] as well as inducing a unique withdrawal syndrome in females [7]. Xylazine further complicates overdose response through prolonged sedation even after breathing is restored by naloxone [8–11]. 

Despite emerging research attempts to study xylazine-related harms for people who use opioids (PWUO) [12, 13], gaps in knowledge about the risks associated with long-term use of xylazine persist, such as the cause of xylazine-associated wounds or the best treatment approaches for xylazine withdrawal. Due to a lack of systematic surveillance of the drug supply, there is limited longitudinal data on how xylazine prevalence has changed over time, as well as co-occurrence with other adulterants (e.g., medetomidine, benzodiazepines). Clinical and health impacts are emerging, such as best practices for wound care and withdrawal protocols. Studies [14, 15] continue to report a growing need to develop wound care strategies to address xylazine-associated wounds and a lack of evidence-based treatment protocols for xylazine withdrawal. Moreover, standard opioid overdose reversal (naloxone) does not counteract xylazine’s effects, requiring supportive care (e.g., airway management, intravenous fluids).

The ever-shifting landscape of the heavily adulterated drug supply [16] [17] requires continuing evaluations and monitoring of xylazine’s clinical significance in the opioid overdose crisis [18]. Research regarding awareness, preference, perception of adverse effects, and changes in withdrawal with xylazine exposure among PWUO is emerging, but additional qualitative work can explore these experiences in greater depth [8, 19]. Specifically, there is a need to understand the impact of xylazine from the perspectives of PWUO, who are directly experiencing the effects of a xylazine-adulterated drug supply. This allows lived experiences and community engagement here to be included in policy and programmatic responses to xylazine.

Philadelphia’s fentanyl supply is heavily adulterated with xylazine. Drug checking results by the Philadelphia Department of Public Health in partnership with the Center for Forensic Science Research and Education in the first two quarters of 2023 found that 99% of fentanyl samples also contained xylazine [20]. This indicates that PWUO in Philadelphia at that time were also using xylazine. Therefore, PWUO in Philadelphia have deep knowledge and experiences with xylazine and unique perspectives to contribute to the existing literature. Our previous research about fentanyl test strips, in which concerns about xylazine and a desire for xylazine test strips arose organically [21], indicates that PWUO in Philadelphia desire tailored harm reduction strategies to avoid xylazine. To date, the United States lacks a robust national harm reduction response to xylazine. There is an urgent need for a rapid response to prevent adverse outcomes for PWUO nationally as xylazine emerges or expands in drug markets in other parts of the country [15] [22]. To that aim, this study engaged PWUO in Philadelphia to explore current experiences with and responses to xylazine adulteration. The findings of this study can help inform the development of person-centered approaches to mitigate the impact of xylazine as it emerges as an adulterant in other markets nationally.

## Methods

### Study design and population

Qualitative methods and results are presented using the Consolidated Criteria for Reporting Qualitative Studies (COREQ) checklist [23]. This was a qualitative study using semi-structured interviews to explore xylazine-related knowledge and experiences of PWUO in Philadelphia. An interview guide was developed to capture the experiences of PWUO with xylazine exposure and how they had attempted to reduce its associated harms. The interview guide was translated into Spanish by a Spanish-speaking study team member (author KMG). We used street intercept in the Kensington neighborhood of Philadelphia, Pennsylvania to recruit participants. We approached people on the street, briefly explained the study purpose, and asked potential participants if they used drugs. Those that self-reported xylazine use were asked if they were available to participate in a one-hour interview about their experiences in a nearby private location. Interviews were conducted one block away in a vacant house rented for research purposes. Participants were eligible for inclusion if they were adults (18 years and older), with past 30-day illicit opioid use, and with suspected xylazine exposure. Exclusion criteria included participants unable to consent as assessed by a team member or unwilling to be audio recorded. We did not systematically collect data on how many people approached were not interested or were ineligible to participate. Participants were compensated with $30 cash for participation. We conducted a total of 40 interviews, two of which were conducted in Spanish. This research was approved by the Institutional Review Board at Thomas Jefferson University.

### Community-based participatory research: Project SAFE

This study used a Community-Based Participatory Research (CBPR) framework in which we included people with living experience of xylazine use in the research process. CBPR is a collaborative research approach that seeks to equitably involve community members, researchers, and/or other relevant stakeholders in the research process, recognizing each stakeholder group’s unique strengths and perspectives [24]. 

For this study, we partnered with the Philadelphia-based organization Project SAFE, which is a collective run by and for people who use drugs and who engage in sex work by delivering harm reduction support to people who use drugs. Their work focuses on outreach to these populations, distribution of harm reduction supplies, and advocacy. Two Project SAFE members with living experiences of opioid and xylazine use were trained in the conduct of human subjects research and qualitative methods. The community researchers assisted with refining the interview guide and recruitment process and conducted qualitative interviews alongside the research team. All interviews with PWUO were co-facilitated by one community researcher (LDJ and GJ, who identify as a transgender woman and cisgender woman, respectively) and one study team member (MKR and KMG, both of whom identify as cisgender women). Community researchers were compensated $20 per hour. A broader group of Project SAFE collective members met regularly with the study team to provide input into all phases of the study. Three collective members interested in authorship co-wrote, reviewed, and edited this manuscript.

### Data collection

We conducted all interviews from January to February 2024. Participant demographics were collected at the time of the interviews. Interviews lasted between 30 and 45 min and were audio-recorded. Members of the academic study team did not have pre-existing relationships with study participants. Community researchers did not interview participants with whom they had a pre-existing relationship. The study team debriefed at the end of data collection days to discuss interview content and observed trends, as well as to discuss emotionally-complex interview topics. Spanish interviews were led by a Spanish-speaking community-researcher (LDJ) and native Spanish-speaking study team member (KMG). Participants were recruited until the team determined that thematic saturation had been reached [25]. 

All data were stored on an encrypted, secure, password-protected server and professionally transcribed using a HIPAA-compliant transcription agency, and then uploaded to NVivo 14 [26]. Spanish interviews (*n* = 2, 5%) were translated into English by bilingual transcribers. Transcripts were reviewed by a native Spanish-speaking member of the research team (KMG) for accuracy. Participants were not re-located to review transcripts.

### Data analysis

Transcripts were reviewed for quality by a team member and then by the entire study team to identify emergent themes [25]. The team employed a collaborative codebook development process. Preliminary codes were developed from the first few transcripts to capture important concepts, and an iterative process was used to establish a final codebook, which was then applied to all remaining transcripts. Analysis was conducted by three coders (RL, TEC, KMG) using a conventional content analysis approach, with 30% of the transcripts double-coded. Inter-rater reliability was ensured using Cohen’s kappa coefficient of > 0.70 for a final kappa of 0.95. Participant demographics were summarized using descriptive statistics. Themes were presented to co-authors from Project SAFE (authors FCG, CG, and AK), who assisted with additional thematic iteration.

## Results

Among 40 participants, 50% were male, 55% White, 32% Latino, with an age range of 25–57 years. Half completed high school and 40% were unhoused. Only nine participants (22.5%) lived in their own house or apartment, with the remaining participants in shelters or staying with family and friends. Gender identity was participant-stated and self-reported. All reported using xylazine and fentanyl, and a majority had experienced an overdose in their lifetime (75%). More than half reported injecting as a preferred method for mode of administration (57.5%), and over a third used more than one method (injecting and smoking [*n* = 10], injecting and snorting [*n* = 3] or snorting and smoking [*n* = 1]) (see Table 1). While a few had more recently started using opioids, most reported using for six or more years (45%). The minimum was less than 1 year, and the maximum was 39 years. The average was 13.16 years and the median was 10 years. Six participants had previously used xylazine test strips.

**Table 1:** Demographics of participants discussing experiences with xylazine in philadelphia, PA (*N* = 40)

Socio-Demographics	N (%)
Age, mean (range), Standard Deviation	37.8 (25–57), 8.27
Gender Identity	
Cisgender male	20(50)
Cisgender female	19(47.5)
Transgender female	1(2.5)
Race ^a^	
White	22(55)
Black	11(27.5)
Asian or Pacific Islander	1(2.5)
Native American or Alaska Native	1(2.5)
Other ^b^	9 (22.5)
Ethnicity	
Hispanic/ Latino	13(32.5)
Education	
Less than High School	13(32.5)
High School grad/ GED	20(50)
Some college	2(5)
2 year degree/Associate's	5(12.5)
Housing	
My own home or apartment	9(22.5)
On the street or in another public place (car, bus, station, etc.)	16(40)
A friend's home	7(17.5)
A family member's home	4(10)
A Single Room Occupancy (SRO)	2(5.0)
A shelter	1(2.5)
In an abandoned house	1(2.5)
Services or programs accessed in the past year^a^	
Substance use services (inpatient, outpatient, methadone, buprenorphine)	32(80)
Prevention Point Philadelphia or another harm	32(80)
reduction program	
HIV/HCV/STI testing	30(75)
Housing/shelters	20(50)
Food bank	17(42.5)
Mental health care	11(27.5)
HIV services	5(12.5)
Job placement or readiness program	5(12.5)
Drugs used in the past 30 days^a^	
Xylazine (*tranq*)	40(100)
Heroin/fentanyl (*dope*)	40 (100)
Crack cocaine (*hard*)	28(70)
Powder cocaine (*coke*)	27(67.5)
Methamphetamine (*speed, tina, crystal, t*)	24(60)
Marijuana/cannabis	20(50)
Benzodiazepines not prescribed to you	13(32.5)
Synthetic cannabinoids (*K2, spice*)	9(22.5)
Prescription opioids not prescribed to you (*Percs, Oxies*)	3(7.5)
Suboxone not prescribed to you	3(7.5)
PCP (*wet*)	2(5)
Methadone not prescribed to you	1(2.5)
Mode of Administration^a^	
Smoking	21(52.5)
Nose/sniffing/snorting	10(25)
Injection	23(57.5)
Involvement with the Carceral System	
None	28(70)
Probation	9(22.5)
Parole	5(12.5)
Overdosed from an opioid (lifetime)	
Yes	30(75)
No	10(25)

^a^ categories are not mutually exclusive

^b^ 7 out 9 identified as Latino

Participants noted profound impacts of xylazine on themselves and the broader community of PWUO in Philadelphia. Conversations primarily centered on four domains of concern: experiences of xylazine-associated wounds on themselves and others, withdrawal experiences marked by intensity of symptoms and a more rapid onset, safety concerns from unconscious sedation, and safety concerns from mobile sedation. Below, we summarize general perspectives of participants about xylazine in the drug market related to general impacts on the community of PWUO in Philadelphia, where they preferred to use xylazine, their mode of administration with xylazine, and changes in overdose patterns. We then elaborate on the four domains noted above. Quotes are presented with participant-selected pseudonyms as well as demographics (age, gender, and race).

### Perspectives about xylazine adulteration of the fentanyl supply

#### Impact on the community

Participants routinely emphasized that PWUO had no choice about whether to consume xylazine with fentanyl compared to fentanyl alone due to an opaque and heavily adulterated drug supply. They commented on the variability of the drug supply, and many expressed a belief that xylazine was in all drugs, not just fentanyl. Xylazine’s impact on the community was severe, with Jenn (39, female, White) noting that many had experienced amputations that she attributed to xylazine-associated wounds: *“Half of Kensington is in wheelchairs*,* with walkers*,* and like people have to get wound care every day”.*

Participants identified xylazine’s prevalence in the drug supply as escalating rapidly during the COVID-19 pandemic. When asked what made them suspect “dope” contained xylazine, most (93%, *n* = 37) described the sedating effects of xylazine, marked by a rapid onset. Some said that xylazine had a distinct taste and smell. Others discussed a burning feeling of xylazine when injected. A few also mentioned different (and sometimes conflicting) observations about xylazine’s appearance, with some describing it as clear when cooked and others describing it as blue, purple, or green.

When asked about the negatives and positives of xylazine, responses varied. Seventeen participants (43%) said that they did not like xylazine at all. Some expressed that they specifically sought it because the drug supply was saturated with xylazine and they became dependent on it. Rachel (34, female, White) said:

*I ended up on tranq because I feel like I was sneak attacked*,* you know what I mean? Because they didn’t tell us that they were switching over from fentanyl to tranq. They just started putting that shit in there. And then all – all the sudden*,* you’re addicted to it.”*

Joseph, 33, male, White, said, *“To be honest*,* I don’t [like xylazine]. But I’m addicted to it. I don’t like it*,* because I didn’t know that that’s what I was using. I thought that it was fentanyl.*

A quarter of participants (25%, *n* = 10) said that they enjoyed using xylazine and did not mention any negatives to using the substance. Reasoning for liking xylazine among this group included the high and sedation associated with xylazine use. Reed (28, male, White) said:

*The thing that is nice is it definitely can make you forget about the problems you’re having… It’s really one of them drugs you take and it washes away time. It washes away. You don’t remember anything. You know*,* you just wake up. That’s why when they say like people have a new level of not giving a fuck*,* it’s the tranq that did that.*

Some participants (33%, *n* = 13) said that while they liked the high and sedation associated with xylazine, there were drawbacks. Some of the negative aspects of using xylazine noted included withdrawal (*n* = 3), vulnerability to robbery or physical safety (*n* = 3), sedation (*n* = 2), a general negative impact on overall quality of life (*n* = 1), wounds (*n* = 1) and all of the above (*n* = 3).

As stated by Shiz (38, female, Black):

*It’s fuckin’ up my life. Sorry for cursin’… I be worried that I might not wake up… I don’t feel like wakin’ up in the morning. I’ve been thinkin’ like damn*,* I got to do this all over again today.*

#### Location of use

Twenty-one participants out of thirty-four (62%) said that they were usually outside when using xylazine. Most participants who used outside did not have the option to use indoors because they did not have a home or safe place to use, or lived somewhere they could not use (i.e., with family) but if they had a choice, would prefer to be indoors. Reasons that participants said they would prefer to use inside if they had the option included not having to use in front of others and protection from vulnerability while sedated. One person (Kevin, 28, male, White) who typically used outside but tried to go indoors when possible said:

*[I use] in the streets usually. Yeah*,* outside. I know a lot of people who have housing*,* so sometimes I go to their houses and do it that way. I hate being around people out here because you can’t like – everyone’s just so […] violent and scheming all the time. Like you know what I mean?*

Only one participant (Brianna, 29, female, White) who usually used outside said it was her preference and explained her reasoning, *“If I use around here*,* I use with people that I know that got – that have my back*,* they can watch out for me and make sure*,* like*,* one*,* I don’t get locked up and*,* two*,* I don’t overdose or somethin’ else happens.”*

Many participants preferred to use xylazine alone in a room with a locked door. They referenced a deep distrust of other people, saying that things had changed since the influx of xylazine into the drug supply in tandem with social fraying due to break-ups of encampments and that robberies and assaults had become commonplace.

#### Mode of administration

To avoid wounds and oversedation, many participants (38%, n = 15)had changed – or attempted to change – their mode of administration away from injecting to either snorting or smoking, and many said they knew others who had done so as well. When in the relative safety of one’s own home, a participant may have chosen to inject to enjoy the full effect of the substance, but if using in public, they may have smoked instead to control the intensity of sedation. Robert (30, male, White), who smoked, said:

*I feel the way I use it is safer. I mean*,* there’s no safe way – there’s nothin’ safe about it*,* but I feel the way that I use it is safer than injecting it only because*,* you know*,* I don’t – I’m in a little more control as far as staying conscious*,* you know what I’m sayin’.*

#### Overdoses

Participants noted an increase in overdoses in the past year and more experiences with being administered naloxone. It was not clear whether “overdoses” were actual overdoses with fentanyl alone, fentanyl overdoses with xylazine on board, or oversedation in which naloxone was unnecessarily administered. Many participants described the experience of overdosing and/or oversedation as traumatic (30%, n = 12). Robert (30, male, White) described trying to determine the distinction between heavy sedation with the tranquilizer and an actual opioid overdose:

*I was just gonna say they’ll stop breathing. Or their breathing will get really spaced out*,* which nine times outta ten is normal for the tranquilizer. Um*,* they’ll turn real pale*,* almost gray in the skin*,* blue in the lips and I hate to say it*,* that’s normal for it. But most people mistake that for an overdose. I mean*,* it’s not normal*,* but it’s normal for a tranquilizer. They almost – they almost look dead and their – their breathing will be so spaced out to the point where you’re like – you’re on the fence like this*,* like should I Narcan ‘em? I know I shouldn’t. I know they’re gonna hate it. They’re gonna be sick when they wake up*,* but should I do it? I don’t wanna – you’re sittin’ there and runnin’ through should I do it*,* should I not*,* you know*,* because a lotta people do get Narcan when they don’t have to be. But*,* uh*,* some people just – you know*,* they’re just bein’ safe. They don’t wanna see nobody die.*

#### Domain 1: wounds

Many study participants identified the wounds associated with xylazine use as being both more prevalent and severe than they were prior to xylazine’s entrance to the drug market. Most participants (75%, 30) had wounds of varying severity, from small blisters or holes with eschar to large wounds covering the majority of calves and forearms. The severity and nature of wounds associated with xylazine use were described extensively by every study participant.

Wounds were thought to vary by mode of administration. One participant who reported snorting xylazine described her experience with a wound beginning to develop, while two other participants described not having wounds because they snorted, but expressed concern about the internal effects the xylazine potentially had on their bodies, with one participant (Jose: 52, male, Latino) sharing *“I’m going through a lot of shit through my nose… you should see the stuff that comes out of there. Oh my god.”* Moya (39, female, Latina) snorted her drugs and reported blowing “*this big ass thing that looked like a piece of bacon*” out of her nose. One participant said that in his experience only people who snorted and shot their drugs got “*tranq burns*,*”* but other participants said wounds could occur with any mode of administration. Injecting into wounds was identified as a common practice, with 11 participants described witnessing it and three participants reporting injecting in or around their wounds.

Wound location was highly variable, with some appearing at injection sites and others appearing elsewhere. In addition to the variability of wound location, it was unclear what placed an individual at risk for developing wounds. Housing status impacted the ability to tend to wounds. Christina (31, female, White) said, *“if you’re homeless*,* especially out in the cold*,* I can’t change [my wound dressings] and stuff like that*,* especially if I don’t have stuff to change it with.”*

Accessing hospital-based care was expressed as a challenge by Brianna (29, female, White), who said, *“I try to go get help*,* and they say it has to be a certain severity. They’re tellin’ me I have to be losin’ my shit in order for them to admit me.”* Alternatively, accessible wound care operated by trusted harm reduction organizations with a long-term presence in the city was cited by many participants as a facilitator to treatment. Although wounds developed rapidly, they were slow to heal, with participants describing wounds that lasted months or years without closing. Marie (36, female, Asian) said, *“It’s been two and a half months and then I have one on my one ankle and one on my leg that’s been since September and they’re not all the way healed.”* Kevin (28, male, White) echoed this experience, “*I have like vicious wounds on both my legs that like I’ve been fighting trying to heal for almost two years now.”* Three participants reported observing maggots living in other people’s wounds.

Open wounds were noted as distinctive both in appearance and in smell – 13 participants (33%) described the strong odor accompanying wounds, saying “*they stink*” of “*rotten flesh*” and “*the smell of death.”* Jenn, who had previously noted the number of people in the neighborhood with xylazine-related amputations, said that someone with wounds could “*clear the whole [subway] off or not [be] allowed on a bus because you stink so bad*.” Wounds were also characterized by many as extremely painful, though two described not finding them painful. Eight participants described the wounds as itchy, with two saying they scratched them until they bled. However, others noted the wounds did not itch at all.

Physically, the wounds themselves were connected by study participants to various adverse health outcomes. Nine participants described how people they knew had received amputations, and two described having fingers that could no longer bend. The wounds also psychologically impacted participants, with several expressing anxiety, fear, or terror at the thought of getting more wounds, getting worse wounds, or having limbs “*fall off*” or be amputated. Some participants (13%, *n* = 5) referred to themselves or others as feeling ashamed or embarrassed by the wounds; two indicated this was a barrier to accessing wound care. There were many other identified barriers to treatment, including a lack of knowledge about how to prevent wounds among PWUO and healthcare providers and a lack of resources to treat them.

#### Domain 2: withdrawal

Participants described xylazine withdrawal as “*terrible*,*” “horrible*,*” “the worst*,*” “so bad*,*”* and “*more intense*” than previous withdrawal experiences from fentanyl or heroin alone. Xylazine withdrawal was also described as lasting “*forever”* and lasting “*longer*” than withdrawal previously, with three participants describing the effects as lasting weeks and one participant describing a case where it lasted months. In addition, nine participants said that withdrawal had a more rapid onset. The sensation of consistently waking up very sick from withdrawal was explicitly mentioned as distinct to xylazine withdrawal by six participants. Kendall (43, female, Latina) reflected:

*Once you wake up and you’ve gone through the whole process of falling asleep while you’re*,* you know*,* standing up*,* once that is over*,* you’re sick again… you know*,* fentanyl*,* at least to me*,* I feel like you get at least about six hours off of it. Tranq is like every two hours*,* it seems like.*

Xylazine withdrawal symptoms varied among study participants but included gastrointestinal complaints, references to brain or audio “zaps,” dizziness, sneezing, extreme fatigue, dry mouth, running nose, and high blood pressure. Eight participants reported gastrointestinal complaints including nausea, vomiting, and/or diarrhea. Two participants described experiencing heart palpitations, one of whom also said xylazine withdrawal led to seizures. Three participants described severe anxiety or panic as being part of the withdrawal experience as well.

Study participants also explored the effects of xylazine withdrawal on behavior and decision making. Some described feeling that the fear of withdrawal was the primary reason they continued to use, despite not experiencing any pleasure or explicitly wanting to stop. Four participants described having to focus their energy on making money or how concern about cost affected their ability to do anything except “hustle” and two described making decisions they believed incurred injection-related risks because of the effects of xylazine withdrawal. Rob (35, male, White) said, *“One rule that I should abide by more*,* that is sometimes I’m like super sick*,* and I can’t find it*,* like should be a little bit more careful on the fucking where I use because that’s just the same as fucking sharing a needle.”*

Participants emphasized that current opioid withdrawal protocols were ineffective or insufficient to treat xylazine withdrawal. *“Even [buprenorphine] and stuff like that*,* nothing. It doesn’t do it. It just doesn’t do it*,*”* said Reed (28, male, White), who had previously discussed enjoyable benefits of xylazine use. Four participants said that in their experience nothing helped with withdrawal symptoms. Joseph (33, male, White) described successive clinical attempts to treat his withdrawal:

*I was on*,* I think*,* 140 milligrams oxy*,* of Oxycodones. Then I was on Dilaudid drip IV. Then I was also on two milligrams of Klonopin because I’ve been prescribed Klonopins years*,* benzos*,* Xanax and Klonopin. They had me one*,* two milligrams twice every six hours*,* something like that. And then every two hours*,* they have me on a 60-milligram instant release Oxycodone*,* and it still*,* I still felt like shit.*

This inadequacy of withdrawal treatment was noted by five participants who said that existing medications for opioid use disorder did not address xylazine withdrawal, and an additional four participants who said nothing they had attempted to date had been successful.

#### Domain 3: unconscious sedation

Participants (85%, *n* = 34) described regularly experiencing deep sedation and waking up with personal belongings missing. Robbery was named as commonplace, with many discussing multiple occurrences. Nicholas (41, male, White) said:

*All my shit’s been stolen. Everything… if I slip up and go to the street*,* I can’t bring my cellphone. I can’t bring jewelry. I can’t bring money. I can’t bring anything because nine times out of ten*,* all your shit’s going to be gone*,* no matter how strong you are.*

Multiple participants discussed times they had stayed with oversedated friends or strangers to protect them and keep them safe. Paul (35, male, White) had witnessed the risks of oversedation in others:

*Yeah*,* you become oversedated and anything can happen to them. Um*,* it’s just like you can’t help it. Like sometimes you might do a little bit and pass out. Like you don’t think you’re going to. But if you do a lot*,* you become over-sedated and like you can do whatever you want to that person*,* smack them*,* kick them*,* rob them*,* and they’re not going to move. So like if a female is out by herself and gets high and passes out*,* like a lot of creeps out here in Philadelphia. So*,* um*,* anything can be their subject*,* anything. I see a lot of like crazy shit going on*.

Participants shared experiences with physical assaults and their concerns about it while using xylazine. They discussed instances when they were physically assaulted by others, as well as people they knew who had been assaulted. Jenn described being attacked in the streets and public transit:

*Just punchin’ on people if they’re just sleepin’. Like*,* I mean*,* it hasn’t – it’s happened to me a few times. Like*,* I’ve gotten*,* like*,* they used to shoot*,* like*,* paintballs and shit at us*,* but – and I gotten slapped in the face a couple times but*,* like*,* they mostly do it to dudes.*

Dominic (37, male, White) described an instance of someone lighting him and his girlfriend on fire while they were sleeping outside at night:

*We were sleeping*,* and somebody lit us on*,* lit us on fire. Um*,* and we woke up*,* and it was already*,* like out of control. Um*,* got out of there. I mean*,* my mind wasn’t clear. It was clear enough*,* thank god*,* because like*,* I didn’t do the amount I usually do*,* thank god*,* because I might not have woken up or I might not have to get out of there.*

Participants expressed that women who used xylazine were particularly vulnerable to sexual assault. As Paul (35, male, White) said:

*I really don’t ask them too many questions because I know it’s traumatizing for them. But I mean I’ve overheard them talking saying they were passed out and they woke up and*,* you know*,* they were raped. They knew they were raped. Their pants were down or whatever the case may be.*

Kendall, who had previously noted xylazine’s rapid effects, described a series of traumatic events she experienced while sedated on xylazine:

*Rape and robbery is something that happens regularly with me. At gunpoint*,* not at gunpoint. You know*,* um*,* and you don’t know at first*,* you know*,* everybody’s so nice in the beginning*,* and then it goes bad really fast because you’re unconscious.…I woke up in the hospital with my pockets cut*,* my $100.00 gone*,* and a lump on my head*,* strapped down. And the last thing I remember was sitting at my next-door neighbor’s house. So that’s what tranq does to me. And I would have never known – I don’t know if he did something to me or not.*

Overall, participants emphasized the difficulty of attempts to stay safer while using xylazine. A few mentioned ways they personally stayed safer, including using less to avoid the sedative effects of xylazine and using with a trusted person who could reposition them while unconscious. Nine participants discussed using or increasing their use of stimulants to counteract the effect of taking xylazine. *“That’s really why I do speedball… just to try to even it out so that if something happens*,* I’m not getting all my stuff stolen and stuff like that.”*, *“I try to do cocaine and crack and the K2 [synthetic cannabinoid] to try to stay up.”*

#### Domain 4: mobile sedation

More than half of participants (*n* = 22, 55%) discussed personal experiences with sedation while being mobile, with some referring to it as “*sleepwalking*” or “*nodding out while walking*”. They shared experiences waking up in other places, neighborhoods, and even other cities. Some described becoming alert while standing in the middle of the street and walking into cars or traffic without being previously aware of their actions. Many expressed that this form of sedation put them at risk of being hit by a car or falling onto the elevated train tracks if they fully lost consciousness while walking, with a few reporting experiencing it firsthand. Rob (35, male, White), previously referenced as experiencing victimization while sedated, said:

*Yeah*,* I fell on the tracks and broke my ribs. Oh*,* my God*,* that was so – and I couldn’t get myself up. Thank God there was people down there and jumped down and pulled me off. I could not – now that was hard*,* like*,* because I was full-blown on the street with no money.*

Kendall said, *“And you’re not able to wake yourself up*,* like*,* you know*,* I’ve fallen and hit my face*,* and still not woken up. Woke up with blood everywhere*,* and that’s how unconscious of a sleep it is.”* Furthermore, participants expressed concerns about not knowing what happened to them after waking up while sedated. For some, the line between mobile sedation and oversedation to the point of losing consciousness was blurred. Jose (52, male, Latino) said, *“It makes you go completely out*,* like not just out of it. And when you come out of your nod some people don’t even know who the hell they are*,* how they got there*,* don’t know what happened. There’s stuff you can’t remember.”*

Many participants spoke of a pressing need for overdose prevention sites to prevent victimization while sedated. Paul (35, male, White) said:

*I see like overseas they’re doing like that [overdose prevention sites]. Like people get high in certain places… maybe legalize it like they do overseas*,* I’ve read. Like where it’s in a controlled environment and with controlled stuff. That’d be like the only way I could see that it would be like done successfully. Otherwise*,* it’s going to keep down the same road that it’s on right now.*

Jack (57, male, White) echoed this belief, saying:

*From my experience is that I would try and work on a situation to where everybody can*,* like*,* do what they have to do in a certain safe environment instead of havin’*,* like*,* God forbid*,* somebody’s like sittin’ there and really wrecked up and somebody stole ten units off ‘em you know what I’m sayin’*,* shit like that. It happens*,* but it’s been happenin’ a lot more and it’s gonna happen more and more.*

## Discussion

Participants in our study had been severely impacted by the presence of xylazine in the fentanyl supply. Most had wounds of varying severity on their bodies at the time of their interview, with these wounds causing pain, disability, and deep shame. They described the distinct agony of withdrawal, differentiating it from prior experiences of withdrawal from fentanyl or heroin alone. Most had experienced unconscious or mobile sedation with repeated robberies and physical injury—several women described experiences of sexual assault.

A greater investment in harm reduction services using a combination of individual and structural-level interventions for people using xylazine is needed. This includes widespread distribution of wound care supplies to prevent theft from others, as well as streetside wound care nursing by trauma-informed clinicians who will not further stigmatize people in need of care. Some participants indicated that they felt too embarrassed by the appearance and odor of their wounds to access care in an indoor setting, underscoring the need to bring services directly to where people are located [27]. Investing in education directly to PWUO about self-management of wounds, coupled with wound care supplies, may be an effective individual-level intervention. Training peers who use xylazine to help treat wounds is another promising potential intervention. Allocating more resources to trusted organizations with an established record of working with PWUO can help ensure a range of people are treated. Mobile services can reach additional people who do not have access to other services from “brick and mortar” organizations or medical settings.

Nearly half of our sample was unhoused, and more still were unstably housed. Both housed and unhoused participants had experienced xylazine-associated wounds. Future studies should explore whether these wounds are more prevalent among unhoused PWUO, due to attending difficulties accessing running water and sterile supplies to dress wounds.

Xylazine withdrawal is increasingly recognized as having distinct physical and psychological effects among people who use drugs when compared to withdrawal from opioids alone [8, 28]. Participants described this withdrawal in detail. Some participants described xylazine’s withdrawal onset as more rapid than fentanyl, which contradicts some earlier literature hypothesizing that xylazine “gives fentanyl legs”, or extends the length of time during which effects from fentanyl are felt [4]. Recent research from the Philadelphia region has identified promising approaches and protocols [15, 29], but more can be learned from listening to the experiences of PWUO. Extensive and ongoing research into effective withdrawal treatment protocols is urgently needed to alleviate suffering, as well as to enable entry into treatment for wounds, other health concerns, and/or treatment for opioid use disorder [15]. Further research is also needed about potential life-threatening complications from xylazine withdrawal, for which emerging evidence is mixed [9, 30]. 

To mitigate the harms of xylazine adulteration in the community, specifically those related to safety, structural interventions for PWUO are crucial. People expressed that they were not in control of the drugs they purchased and consumed and regularly noted the futility of attempts to protect themselves from robbery or other harms of an unpredictable drug that could cause profound sedation. Point of care drug checking is an important service that can help people understand the composition of the drug supply and what is in a sample they provide to a program. Studies have demonstrated that PWUO would use services to check for xylazine [31, 32]. Whether for avoiding or detecting xylazine, such a service is needed to provide more control and navigation over the hard-to-predict drug supply. Since so many participants indicated a need to use xylazine to stave off withdrawal, xylazine seeking as well as xylazine alternatives are both realistic adaptations to the unpredictable illicit market and drug-related harms. Test strips, which had previously been identified in Philadelphia as a desired harm reduction tool before they were available [21], are increasingly available in Philadelphia and elsewhere [33, 34]. However, research conducted with PWUO after xylazine fully saturated the Philadelphia market indicated that test strips would no longer be helpful given the need to use xylazine to avoid withdrawal [35]. We suggest more effective would be a safer supply of opioids, coupled with effective xylazine withdrawal management, to help people stay safer while using. Safer supplies of opioids can be provided through different programmatic structures, from prescribed forms that are taken under supervision, programs in which participants can take their supply home, or hypothetically a supply under government regulation to ensure a product of free of harmful adulterants [36, 37]. 

Other structural interventions, such as overdose prevention sites, are urgently needed in all communities, including those experiencing xylazine adulteration of the opioid supply. Participants referenced wanting a safe place to use xylazine. For many, this safety was stated as using alone and behind a locked door, meaning people were trying to balance overdose prevention and physical safety. Decades of research indicates that an overdose prevention site could offer a safe place for people to use and recover and to be linked to wound care and other services as desired [38]. Further, these sites would provide a safe space for participants during heavy sedation after use and would allow for ongoing monitoring. Interventions should be developed through partnerships of people who use drugs and other content experts working closely with them.

To our knowledge, this is the first paper to provide in-depth information about mobile sedation of people using xylazine, building on preliminary work from our team [21, 35]. A focus on the understudied outcomes of xylazine use is essential as it presents risks for severe injury and psychological harm. Many interventions are not well-positioned to address this aspect of xylazine use. A safer place to use and rest after using substances, if welcoming and comfortable, may encourage PWUO to stay in a safe space while sedated but mobile.

Our study was subject to several limitations. Participants were recruited from a city with greater xylazine adulteration than is currently found elsewhere in the world. Their experiences of xylazine dependence and withdrawal may not be a reality for other PWUO. This is also a strength of the study because there are important lessons for other locations where xylazine continues to spread as an adulterant. Further, our sample represented very structurally vulnerable PWUO as many were unhoused or unstably housed and may not represent the experiences of all PWUO. Still, we believe their experiences have important implications for other locations experiencing xylazine adulteration of fentanyl. More research is needed on xylazine, including domains presented here, to develop effective individual and structured interventions to address xylazine wounds, sedation, and withdrawal.

Our study also includes the strength of partnering with people currently using opioids to collect data. We believe that interviews led by people with shared positionalities [39] of xylazine use fostered rapport and trust during the interview process, culminating in a rich dataset.

## Conclusion

Xylazine has had many impacts on PWUO, including severe wounds, withdrawal, and sedation, leading to increased risk of robbery, physical or sexual assault, or accidents while sedated but mobile. Individual attempts to reduce these harms must be coupled with addressing structural factors driving these risks. Interventions should be developed through partnerships of people who use drugs and other content experts working closely with them. Higher-level policy and programmatic changes are critical to reducing the harms associated with xylazine use.

## Data Availability

Aggregated, de-identified data can be provided by the corresponding author on a case-by-case basis.

## Abbreviations

PWUO: People who use opioids
